# Haloperidol effects on striatal dopamine and DOPAC levels and subcellular distribution

**DOI:** 10.1186/1471-2202-13-S1-P15

**Published:** 2012-07-16

**Authors:** Lane J Wallace

**Affiliations:** 1 Division of Pharmacology, College of Pharmacy, Ohio State University, Columbus, OH 43210, USA

## 

Signaling involving dopaminergic neurons in the brain is important for determining the saliency of sensory input, the urgency of sensory import, the actual value of an experience as compared to its expected value, and the process of converting motivation to action. Drugs that are antagonists for the dopamine D2 receptor subtype are clinically effective in treating schizophrenia and a variety of other mental conditions. These drugs have a substantial effect on the levels and sub-cellular distribution of dopamine and its major metabolite DOPAC. Currently, there is no quantitative analysis of how these various changes can be accomplished. The goal of this work is to explain how the changes in dopamine and DOPAC levels and subcellular location elicited by the prototypical antipsychotic drug haloperidol occur.

A computational model of a dopaminergic striatal varicosity was used. In the model, dopamine can be located in cytosol, vesicles, extracellular compartment, and a DOPAC-synthesis-secretory compartment. DOPAC can be located in cytosol, extracellular, and DOPAC-synthesis-secretory compartment. The model has two distinct sites for dopamine and DOPAC synthesis. The cytosolic site synthesizes dopamine for use in the signaling pool and metabolizes a portion of dopamine that is transiently located in cytosol. The DOPAC-synthesis-secretory complex site is hypothesized to be located on the plasma membrane and to be dedicated to the synthesis of DOPAC to be secreted into the extracellular compartment. The model also has enzymes involved in dopamine and DOPAC synthesis, transporters that move dopamine between compartments, and exocytosis.

Haloperidol induces a small increase in extracellular dopamine, a somewhat larger increase in extracellular DOPAC, a larger increase in tissue DOPAC, a large increase in rate of dopamine synthesis, and no change in level of dopamine. The increase in extracellular dopamine was simulated by increasing rate of secretion of dopamine into extracellular space by exocytosis. An increase in synthesis of signaling dopamine was required to maintain the elevated levels of extracellular dopamine. The increase in extracellular DOPAC was simulated by increasing rate of dopamine synthesis in the DOPAC-synthesis-secretory compartment. With only these manipulations, the model failed to match experimental data in terms of increase in total tissue DOPAC and increase in the rate of dopamine synthesis. These discrepancies were resolved by increasing the passive diffusion of dopamine from vesicles to cytosol and further increasing the rate of dopamine synthesis for the signaling pool. Literature data indicate that haloperidol does accumulate in and partially alkalinize amine neurotransmitter storage vesicles, which would increase passive diffusion of dopamine from the vesicles.

I conclude that the increases in extracellular dopamine and DOPAC are likely mediated by antagonism of dopamine D2 receptors but that the large increase in tissue DOPAC and much of the increase in dopamine synthesis result from physico-chemical properties of haloperidol rather than block of dopamine D2 receptors. Furthermore, the results suggest dopaminergic varicosities possess a mechanism for monitoring amount of stored dopamine and modulating dopamine synthesis to maintain constant stores.

